# An Environmental Scan of Recent Initiatives Incorporating Social Determinants in Public Health

**DOI:** 10.5888/pcd13.160248

**Published:** 2016-06-30

**Authors:** Denise Koo, Patrick W. O’Carroll, Andrea Harris, Karen B. DeSalvo

**Affiliations:** Author Affiliations: Patrick W. O’Carroll, Rear Admiral, Assistant Surgeon General, Regional Health Administrator, US Public Health Service Region X, Office of the Assistant Secretary for Health, US Department of Health and Human Services, Seattle, Washington; Andrea Harris, Chief of Staff, Office of the Assistant Secretary for Health, US Department of Health and Human Services, Washington, DC; Karen B. DeSalvo, Acting Assistant Secretary for Health, US Department of Health and Human Services, Washington, DC.

**Editor’s Note:** This article is a joint publication initiative between *Preventing Chronic Disease* and the National Academy of Medicine.

## Cross-Sector Collaboration to Improve Public Health

The foundational importance of social, environmental, and economic factors as determinants of health has long been recognized (1–5). Until recently, this recognition had resulted in few sustained, organized efforts to positively influence these determinants to foster health at the community level. In recent years, however, numerous efforts have arisen across the United States that explicitly seek to improve the public’s health by catalyzing collaboration across multiple societal sectors, with the goal of leveraging policy, systems, and environmental changes to drive sustained improvements in the public’s health. Many are using concepts such as “Health in All Policies” (6,7) and collective impact (8) to structure their efforts. These initiatives vary in scope and scale, and they address the challenge of multisector approaches to the social determinants in a variety of ways, often innovating as they evolve.

These pioneering efforts face several challenges. First, they are generally not guided by any national strategy or coherent plan to promote such cross-sector partnerships or to leverage resources across these initiatives. Second, many community efforts are developing in relative isolation, with little opportunity to learn from the successes and failures of similar undertakings in other communities. Third, the accelerated pace at which broad, health-oriented community collaboratives are being launched makes it difficult to maintain awareness of the many opportunities, toolkits, and frameworks that already exist.

These efforts are laudable and offer much to adapt and apply in communities across the country. To begin to systematize such cross-sector, expansive approaches to community health, in this article we identify, categorize, and describe an array of multisector initiatives and collaborations currently under way across the United States that explicitly include attention to social, economic, and environmental factors to foster community health and well-being.

## Achieving a Coordinated Cross-Sector Effort

We sought to identify initiatives in the United States with the following characteristics: 1) an explicit (though not necessarily sole) goal of promoting health and well-being, distinct from improving health care; 2) a holistic definition of health; 3) an effort to address a broad set of social, environmental, or economic drivers of health; and 4) active involvement of government and nongovernment partners from at least 2 sectors (eg, public health, health care, housing, transportation, city planning, education, food systems, parks).

Between June 2014 and December 2015, we gathered information about these initiatives through an iterative process. We participated in national public health and health care meetings; discussed initiatives with leaders focused on population health, public health, or community health; and reviewed key national reports, newsletters, and websites. Our sources were Academy Health, American Hospital Association’s Association for Community Health Improvement, American Public Health Association, America’s Essential Hospitals, Association of Academic Health Centers, Association of American Medical Colleges, Association of State and Territorial Health Officials, Build Healthy Places Network, Catholic Health Association, Institute for Healthcare Improvement, National Academies of Sciences, Engineering, and Medicine, National Association of City and County Health Officials, National Network of Public Health Institutes, National Quality Forum, Prevention Institute, Public Health Accreditation Board, Robert Wood Johnson Foundation, and VHA, Inc, now part of Vizient, Inc. We also identified initiatives by conducting a systematic review of tools designed to support community health and by searching the Web for the terms “population health,” “community health,” “health system transformation,” “public health and health care integration,” and “social determinants,” along with the terms “movements,” “tools,” and “resources.” Given the rapid pace at which new initiatives and tools are being launched, the set of initiatives we identified should be considered broadly representative and not comprehensively inclusive.

## Types of Initiatives

Our research yielded hundreds of initiatives to address social determinants, a large number with health as a primary goal. These initiatives varied widely in scope, scale, and approach. Many initiatives we identified began in the health sector, but several were introduced by other sectors and included health as one of several targeted outcomes.

Most of these initiatives fell into 6 categories: 1) community-generated initiatives to foster community health; 2) data and metrics initiatives to support measurement of community health; 3) toolkits to promote multisector efforts to promote health; 4) campaigns intended to inspire broad multisector approaches to health; 5) federal initiatives promoting a broad vision for fostering community; and 6) philanthropic initiatives supporting and motivating multisector collaboration to improve health. 

### Community-generated initiatives

We identified more than 100 locally led initiatives that focused on crosscutting approaches to the health of the community or, more broadly, on the economic viability and livability of the city or county, including the public’s health. These community-led initiatives were found across the country and involved the public health and health care sectors working in concert with other sectors that affect social determinants such as housing, transportation, social services, and other local government and community organizations. Health-centric initiatives such as Live Well Sioux Falls, Healthy Chicago, or Healthy Living Matters (Harris County, Texas) are often led by the local health department or a local coalition that involves the health department and other partners. Other initiatives like Live Well San Diego, Mayor’s Healthy City Initiative (Baton Rouge, Louisiana), and Healthy Riverside County (California), which strive to make their communities safe, thriving, *and* healthy, are typically spearheaded by the executive leader of the government, whether the mayor, governor, or county leader, and frequently involve the local health officer in a critical leadership role. As in many other cities, San Antonio, Texas, has incorporated a nonprofit “backbone” organization, SA2020, to coordinate coalition efforts to transform San Antonio into “a world-class city by 2020” (www.sa2020.org). Lists of community collaboratives that won community health prizes, participated in learning collaboratives, or were supported by nonprofit health organizations illustrate the variety of bottom-up initiatives led by community organizations across the country (9–17). The ReThink Health 2014 Pulse Check survey, in particular, identified 133 multisector partnerships for health, with the health care (n = 123) and public health (n = 119) sectors most commonly represented among the partners engaged (18). Communities with partnerships of the longest duration — and frequently the greatest success — often appear on multiple lists.

### Data and metrics initiatives

Data and metrics play a crucial role in setting goals and measuring the health of communities. We define metrics as data-driven goals for assessing a community’s health and for measuring progress toward such. We identified 7 major national crosscutting health-focused metrics efforts that explicitly included social determinants of health and 2 healthy or livable community indices, one developed by the US Department of Housing and Urban Development (HUD) and another by AARP, Inc (formerly the American Association of Retired Persons) (Table 1). Each crosscutting health metrics initiative is intended to guide improvement in the health of the community, county, state, or the nation, and includes at least one metric for mortality, well-being, health behaviors, clinical care, physical environment, and socioeconomic factors. The HUD and AARP indices list health as only one domain for measurement and also include environment, transportation, housing, neighborhood, social engagement, and economic opportunity.

**Table 1:** National Metrics Initiatives Supporting Community Health, United States, June 2014–December 2015

Name	Organization(s) involved	URL
Healthy People 2020: Leading Health Indicators	Office of Disease Prevention and Health Promotion, Office of the Assistant Secretary for Health, US Department of Health and Human Services	http://www.healthypeople.gov/2020/Leading-Health-Indicators
National Prevention Strategy	Office of the Surgeon General, Office of the Assistant Secretary for Health, US Department of Health and Human Services	http://www.surgeongeneral.gov/priorities/prevention/strategy/index.html
America’s Health Rankings	United Health Foundation, Partnership for Prevention, American Public Health Association	http://www.americashealthrankings.org/
County Health Rankings and Roadmaps	University of Wisconsin, funded by Robert Wood Johnson Foundation	http://www.countyhealthrankings.org
Community Health Status Indicators	Centers for Disease Control and Prevention	http://wwwn.cdc.gov/CommunityHealth/
Vital Signs: Core metrics for health and healthcare progress	Institute of Medicine	http://iom.nationalacademies.org/Reports/2015/Vital-Signs-Core-Metrics.aspx
Measures to Mobilize a Culture of Health	Robert Wood Johnson Foundation	http://www.cultureofhealth.org/content/dam/COH/FromVisiontoActionMeasuresCompendium2016.pdf
Healthy Communities Assessment Tool	US Department of Housing and Urban Development	Tool used at http://hcat.providenceri.com/
AARP Livability Index	AARP	http://aarp.org/livabilityindex

### Toolkits

Many resources promote and support multisector efforts to improve population health by providing a vision for the health of the community, practical tools to aid in its achievement, and examples of others’ successes. We defined *comprehensive* resources as those comprising 1) a conceptual model or theory of change for improving the community’s health, 2) a suggested set of actions or steps to improve community health, 3) resources to support collaboration with other sectors, and 4) examples of successful collaborative partnerships to improve health. We found 6 toolkits that fit these criteria (Table 2). We included only tools targeting more than one sector and freely available on the World Wide Web. Several instances of the comprehensive tools are also associated with prizes for collaborative work, technical assistance or coaching, training, webinars, blogs, and links to data sets and rankings.

**Table 2:** Comprehensive Toolkits Supporting Community Health, United States, June 2014–December 2015

Name	Lead Organization	Primary Audience	Features Unique To Toolkit	URL
Build Healthy Places Network	Build Healthy Places Network	Health and community development sectors	Logic models for various health conditions; MeasureUp (mapping and measurement tools); community close-ups that highlight role of community development	http://buildhealthyplaces.org/
Community Commons	IP3 and CARES-University of Missouri	Broad	Access to and ability to visualize social determinants data in graphs, maps, and other formats; content from the field organized in “channels,” including economy, education, environment, equity, food, and health; houses “hubs” where organizations, initiatives, and collaboratives can share content, data, and resources	http://www.communitycommons.org/
Community Health Improvement Navigator	Centers for Disease Control and Prevention	Hospitals, public health sector, community partners	Community Health Improvement Infographic; key quotes from Internal Revenue Service final rule on Community Health Needs Assessments for Charitable Hospitals; search engine for evidence-based community interventions	http://www.cdc.gov/chinav
Community Toolbox	University of Kansas	Broad	Online training, curriculum, community workstations; materials in multiple languages; troubleshooting guide; guestbook to describe use of toolbox	http://ctb.ku.edu/en
County Health Rankings and Roadmaps Action Center	University of Wisconsin Population Health Institute	Broad, community partners	County Health Rankings; What Works for Health database; model of population health; partner guides (including for public health)	http://www.countyhealthrankings.org/
Practical Playbook	Duke University School of Medicine, Department of Community and Family Medicine	Public health sector, primary care providers	Similar content also published as a textbook: The Practical Playbook: Public Health and Primary Care Together. JL Michener, D Koo, BC Castrucci, JB Sprague, editors, New York (NY): Oxford University Press, 2016; first national meeting May 2016	http://practicalplaybook.org

### Campaigns

An increasing number of initiatives have been launched with a goal of inspiring multisector approaches to improving community health. We categorized these efforts as campaigns, which we define as a group of people or organizations working together to advance their shared ideas for improving the health or vitality of a community. The 11 campaigns identified present compelling arguments for supporting a multisector approach to health; they encourage others to join their effort, and often offer newsletters for maintaining contact as well as tools to enhance their work (Table 3). These health campaigns implicitly or explicitly include fundamental determinants of health such as education, nutrition, and environment and emphasize the need to address such determinants in partnership with other sectors in communities. Seven of these 11 campaigns target audiences in the health care system (eg, primary care providers, hospitals, academic health centers).

**Table 3:** Health Campaigns Supporting Community Health, United States, June 2014–December 2015

Name	Goal	Lead Organization	Primary Target Audience	URL
Generation Public Health	Healthiest nation in one generation	American Public Health Association	Public health sector and others	http://www.apha.org/healthiest-nation
Culture of Health	Creating a culture of health	Robert Wood Johnson Foundation	Broad	http://www.cultureofhealth.org/en.html
Integration Forum (primary care and public health collaborative)	Accelerate integration that improves population health and lowers costs	Association of State and Territorial Health Officials	Public health sector, primary care providers	http://www.astho.org/Programs/Access/Primary-Care-and-Public-Health-Integration/
Build Healthy Places Network	Catalyze and support collaboration across health and community development sectors	Build Healthy Places Network	Public health sector, health systems, community development	http://www.buildhealthyplaces.org/
100 Million Healthier Lives	100 Million people living healthier lives by 2020	Institute for Healthcare Improvement	Broad	http://www.100mlives.org/
Social determinants of health movement	Facilitating ability of academic health centers to address determinants of health	Association of Academic Health Centers	Academic health centers	http://www.aahcdc.org/Resources/SocialDeterminantsofHealth.aspx
Health Begins	Move health upstream, improving care, addressing social determinants and health equity	Health Begins	Primary care providers	http://www.healthbegins.org/
Stakeholder Health	Address underlying causes of poor health	Stakeholder Health	Hospitals, health systems	http://stakeholderhealth.org/
Moving Healthcare Upstream	Address health disparities that originate in the early years of life	UCLA/Nemours, funded by Kresge Foundation	Health systems	http://movinghealthcareupstream.org/
Beyond Flexner Alliance	Integrate social mission into health professions education and practice	George Washington University	Health professions education	http://beyondflexner.org
Health is Primary	Build primary care system that puts patients at center and improves health	Family Medicine for America’s Health	Created on behalf of family physicians for the public	http://www.healthisprimary.org/

Abbreviation: UCLA, University of California Los Angeles.

### Federal initiatives

The federal government has launched a variety of initiatives aimed at promoting a broad approach to community health (Table 4). Many are sponsored by the US Department of Health and Human Services (DHHS). For example, in 2015 DHHS released the Community Health Improvement Navigator (www.cdc.gov/chinav), and the Office of the Assistant Secretary for Health inaugurated Public Health 3.0 (19). In 2016, DHHS issued a funding opportunity announcement for Accountable Health Communities (https://innovation.cms.gov/initiatives/AHCM). Similar or complementary initiatives linking health and housing, transportation, or community development have also been launched by HUD, the Department of Transportation, and the Federal Reserve, respectively. These national initiatives have several characteristics in common: they acknowledge the relevance of health and the health sector to the mission of other disciplines and vice versa, they include health as a primary or at least a relevant goal, they underscore the importance of collaboration across disciplines, and they support or incentivize such multisector partnerships.

**Table 4:** Examples of Federal Agency Initiatives Supporting Community Health, United States, June 2014–December 2015

Lead Organization	Description	URL
Corporation for National and Community Service	With the simple but vital goal of finding what works, and making it work for more people, the Social Innovation Fund and its grantees create a learning network of organizations working to implement innovative and effective evidence-based solutions to local and national challenges in 3 priority areas: economic opportunity, healthy futures, and youth development.	http://www.nationalservice.gov/programs/social-innovation-fund
US Department of Agriculture	Promise Zones are high-poverty communities where the federal government partners with local leaders to increase economic activity, improve educational opportunities, leverage private investment, reduce violent crime, enhance public health, and address other priorities identified by the community. (Partnership between HUD and USDA.)	https://www.hudexchange.info/programs/promise-zones/
US Department of Health and Human Services	HHS has numerous initiatives supported by agencies across the department: Accountable Health Communities, Community Health Improvement Navigator, Healthy People 2020, Public Health 3.0, National Prevention Strategy, Partnerships to Improve Community Health, State Innovation Models.	http://www.hhs.gov
US Department of Housing and Urban Development	HUD has numerous healthy housing and healthy communities initiatives: Lead Hazard Control and Healthy Homes Program and the Healthy Communities Transformation Initiative, with its Healthy Communities Assessment Tool and Healthy Community Index. Also a partner with EPA and DOT on Partnership for Sustainable Communities.	•http://portal.hud.gov/hudportal/HUD?src=/program_offices/healthy_homes/hhi •http://hcat.providenceri.com
US Department of Transportation	Transportation and Health Tool facilitates examination of the impact of the transportation environment on health and identification of strategies to improve public health through transportation planning and policy. Also a partner with EPA and HUD on Partnership for Sustainable Communities.	https://www.transportation.gov/transportation-health-tool
US Environmental Protection Agency	The Human Well-being Index includes a health dimension to support decisions that contribute to the sustainability of built and natural environments (26). Also a partner with HUD and DOT on Partnership for Sustainable Communities.	https://www.sustainablecommunities.gov/
Federal Reserve	The Federal Reserve supports collective approaches that involve building quality housing, educational systems, job programs, transportation, and community wellness organizations. The Healthy Communities Initiative was designed to enrich the debate on how cross-sector and place-based approaches to revitalize low-income communities might both revitalize neighborhoods and improve health.	Dallas and San Francisco Federal Reserve banks are especially active: •http://www.dallasfed.org/cd/healthy/index.cfm •http://www.frbsf.org/community-development/initiatives/healthy-communities/

Abbreviations: DOT, US Department of Transportation; EPA, US Environmental Protection Agency; HHS, US Department of Health and Human Services; HUD, US Department of Housing and Urban Development; USDA, US Department of Agriculture.

### Philanthropic initiatives

We identified scores of initiatives supported by local and national foundations of various sizes and missions (health- and nonhealth–related), acting both independently and jointly. Many small and large health and health care foundations encourage multisector partnerships for health improvement among public health, health care, and community, or among health and community development, often through prizes, grants, or technical assistance. For example, the Robert Wood Johnson Foundation (RWJF), through its focus on a Culture of Health, supports many of the metrics, tools, and campaigns described in this article, as well as prizes for communities. Other foundations not focused on health or public health also support multisector approaches to health either directly or indirectly, given their own focus on health equity (Kresge Foundation) or on supporting children (Kellogg Foundation).

Several major foundations and health care institutions also came together in 2006 to form the Convergence Partnership (http://www.convergencepartnership.org/) and the Convergence Network of more than 80 local and regional funders, working together to foster healthier and more equitable environments for all children and families. The US Chamber of Commerce Foundation partnered in 2015 with RWJF to launch a “Health Means Business” initiative, which includes Healthy10 awards to recognize “cross-sector partnerships with a business-led component that are leading the way to healthier communities” (https://www.uschamberfoundation.org/better-health-through-economic-opportunity/get-involved). These examples provide a snapshot of the range and level of foundation support for multisector collaboration to build healthy, safe, and thriving communities.

## Addressing the Complex Issues Surrounding Public Health

A large and growing array of initiatives underway aims to improve community health by explicitly addressing social, economic, and environmental determinants of health. In this article we describe and categorize a variety of multisector initiatives in this arena, unified by an “upstream” approach to community health. Although there is some overlap among our categories of data and metrics initiatives, toolkits, campaigns, and federal programs, the initiatives and references highlighted here represent only a minimum estimate of the number of activities throughout the United States, especially at the community level. Of note, the leadership and energy for this crosscutting approach to community health comes both from health (public health and health care) and nonhealth sectors, and these initiatives originate both bottom-up from community leaders as well as top-down from all levels of government, national organizations, and philanthropies.

This convergence of crosscutting national initiatives from different sectors, both with each other and with community-driven efforts, is a welcome and critical development. Despite great progress in health care access and quality over the past few years and great progress in both public health science and organizational capacity in the last century, the health status of Americans is still far below where it needs to be and where it can be (20–24). At this juncture major new improvements in health will require societal action that goes beyond the traditional work of the public health and health care sectors. This requirement is reflected in the Institute of Medicine’s definition of public health as “the collective actions of a society to ensure the conditions in which all people can be healthy” (2).

Indeed, the vision for health promulgated by the initiatives described in this article acknowledges that “creating places where people want to live . . . [involves ensuring that such are places] where it is healthy to live” (25), and the vision requires multisector collaboration. The campaigns described build momentum for initiating the cross-sector changes and approaches necessary to address the social determinants and improve the conditions for achieving health and well-being. The metrics initiatives, by their inclusion of measures from a variety of sectors, reinforce the need for multisector collaboration. America’s Health Rankings and the County Health Rankings have particularly spurred multisector efforts at the state and county level to become the “healthiest” in the nation or the state. The many and varied toolkits available provide ample opportunity for community stakeholders to identify one or more approaches that fit their needs. Foundation-based initiatives help catalyze innovation in this complex arena, and the federal government helps to institutionalize it. Most importantly, however, these efforts together enhance the capacity of communities to build their own healthy future.

Kania and Kramer (8) described the model of *collective impact* for addressing “our most serious and complex social problems.” They identified improving community health as a challenge requiring the collective impact of nonprofits, government, business, and the public working together on a common agenda. On the basis of their research, they identified 5 conditions for collective success: common agenda, shared measurement system, mutually reinforcing activities, continuous communication, and a backbone support organization. The broad, community efforts for public health exemplified by the initiatives described in this article serve as strong models of collective action to improve the conditions in which all people can be healthy. These initiatives identified *a common agenda* across sectors (healthy, thriving communities), with generally consistent crosscutting approaches to *metrics* **,** and *activities across the initiatives that are mutually reinforcing*. Given the rapid pace of innovation in this space, there is a need for greater *communication* and sharing among these initiatives, facilitated by the support and coordination of a *backbone sector* such as public health.

The window has opened for sustainable change. To leverage this critical opportunity, we need to coordinate our multiple, varied efforts to minimize duplication of effort and to maximize impact on the health of the public. Public health agencies at local, state, and federal levels, in partnership with other government leadership, can play a vital role in leading across silos and bringing the various sectors together. Although each community will have distinct objectives and strategies for achieving them, public health’s role in the community-driven multisector approach can be systematized and organized around several key components: enhanced leadership and workforce; new partners; accreditation and foundational public health services; data, metrics, and analytics; and appropriate funding. This systematized approach has been referred to as Public Health 3.0 (19 *)*. The essence of the Public Health 3.0 framework is multisector collaboration that leverages social determinants to improve the health of communities, with public health agencies at the center. The time is ripe for a collective impact approach to improving the public’s health; the time is ripe for Public Health 3.0.
